# CFTR Modulators Counteract F508del CFTR Functional Defects in a Pancreatic Epithelial Model of Cystic Fibrosis

**DOI:** 10.3390/life15081315

**Published:** 2025-08-19

**Authors:** Alessandra Ludovico, Debora Baroni

**Affiliations:** Istituto di Biofisica, National Research Council (CNR), Via De Marini, 6, 16149 Genova, Italy; ale.ludo89@gmail.com

**Keywords:** cystic fibrosis, CFTR modulators, pancreatic duct epithelial model, inflammation, CFPAC-1 cells

## Abstract

Cystic fibrosis is a multisystem disorder caused by mutations in the *CFTR* gene that lead to impaired ion and fluid transport across secretory epithelia. Although the therapeutic impact of CFTR modulators has been extensively studied in airway epithelia, their efficacy in extra-pulmonary tissues, such as the pancreas, has been less explored. This study evaluated the effects of the CFTR modulators, VX770 (ivacaftor), VX661 (tezacaftor), and VX445 (elexacaftor), administered either individually or in combination, on CFPAC-1 cells, a pancreatic ductal epithelial cell line derived from a cystic fibrosis patient harboring the F508del CFTR mutation. The cells were cultured and differentiated onto porous supports, and a panel of functional parameters was assessed. These included transepithelial electrical conductance, fluid reabsorption, apical surface fluid pH, protein concentration, and microviscosity, the latter analyzed with multiple particle tracking. To simulate a pro-inflammatory micro-environment, the cells were preconditioned with lipopolysaccharide (LPS). Treatment with VX661 and VX445 resulted in significant improvement in epithelial function, with the triple combination producing the most pronounced rescue. Pro-inflammatory stimulation by LPS increased the production of cytokine IL6, IL-8, and IL-1β, as well as the protein content of the apical surface fluid. Despite the LPS pro-inflammatory stimulus, CFTR modulators preserved or slightly enhanced their efficacy in restoring CFTR-mediated ion and fluid transport. However, they did not reduce cytokine expression under pro-inflammatory conditions. Collectively, these findings show that CFTR modulators can restore critical aspects of cystic fibrosis pancreatic epithelial physiology in vitro, even under pro-inflammatory stress, supporting their potential relevance beyond the airway disease.

## 1. Introduction

Cystic fibrosis (CF) is the most common fatal autosomal recessive disease in Caucasian populations, affecting approximately one in 2500 live births in Europe and North America [1]. It is caused by mutations in the gene that encodes the cystic fibrosis transmembrane conductance regulator (CFTR), which is a cAMP-activated chloride (Cl^−^) and bicarbonate (HCO_3_^−^) channel located on the apical membrane of epithelial cells [2]. To date, more than 2000 CFTR mutations have been identified. The most common is the deletion of phenylalanine at position 508 (F508del), which is present in more than 90% of patients [3].

CF is a multisystem disease, with major clinical manifestations affecting the lungs, pancreas, gastrointestinal tract, and reproductive system [4]. In the airways, CFTR dysfunction leads to impaired mucociliary clearance, persistent bacterial infections, chronic inflammation, and progressive bronchiectasis, ultimately resulting in respiratory failure [5]. In the pancreas, CFTR dysfunction leads to the dehydration of pancreatic secretions, ductal obstruction, inflammation, fibrosis, and pancreatic exocrine insufficiency in almost 85% of patients. Patients categorized as pancreatic insufficient (PI) typically have a more severe disease phenotype than the remaining 15%, who are pancreatic sufficient (PS) [6,7].

The F508del mutation impairs the folding and trafficking of CFTR, leading to its retention in the endoplasmic reticulum and subsequent degradation via the ubiquitin-proteasome pathway [8]. However, if the F508del CFTR protein is properly trafficked to the apical membrane, it retains partial function [9,10]. This observation has guided the development of pharmacological strategies combining correctors, which improve CFTR folding and trafficking, with potentiators, which enhance channel activity. The most successful of these therapies is Kaftrio/Trikafta: a triple combination of the potentiator ivacaftor (VX770) with the correctors tezacaftor (VX661) and elexacaftor (VX445). This combination has demonstrated substantial clinical benefits for patients carrying at least one F508del allele, including improved lung function, reduced sweat chloride concentration, and enhanced quality of life [11,12].

CFTR is highly expressed in the pancreatic ducts, where it regulates the secretion of fluids and electrolytes, particularly bicarbonate (HCO_3_^−^) [13,14,15]. Bicarbonate secretion is crucial for solubilising mucins and digestive enzymes, as well as maintaining the optimal viscosity of pancreatic secretions [16,17]. As early proposed by Quinton [18,19], the lack of bicarbonate prevents the normal mucin unfolding due to inadequate Ca^2+^ and H^+^ ion sequestration, resulting in abnormally dense and sticky mucus. More recent evidence has confirmed that bicarbonate-driven chelation of divalent cations is essential for the expansion and proper unfolding of secreted mucins, a process fundamental to mucus hydration and clearance in CF [20,21,22,23]. This pathological cascade may contribute to epithelial damage, inflammation, progressive fibrosis, and, ultimately, pancreatic injury [24].

Although inflammation is a hallmark of CF pathology, the response of the CF pancreas to pro-inflammatory stimuli remains comparatively understudied. Unlike in the lungs, where neutrophilic inflammation and excessive cytokine release drive progressive tissue damage, pancreatic inflammation in CF is thought to involve more subtle, yet chronic, epithelial activation, innate immune signaling, and cellular stress responses [25,26]. Toll-like receptors (TLRs), nucleotide-binding oligomerization domain-like receptors (NLRs), and inflammasome components have been identified in pancreatic ductal cells. Here, they respond to pro-inflammatory stimuli and contribute to the production of cytokines, including IL-6, IL-8, IL-1β, TNF-**γ**, and TNF-α [27]. These cytokines may contribute to exacerbating epithelial dysfunction, ultimately worsening exocrine pancreatic insufficiency [28,29].

Notably, stimulation with lipopolysaccharide (LPS), a component of Gram-negative bacterial membranes and a known ligand of TLR4, induces the release of pro-inflammatory cytokines in various epithelial models, including airway and intestinal epithelia [30,31,32]. Consequently, LPS exposure is widely used to reproduce epithelial inflammatory stress in vitro. Although the pancreas is generally considered a sterile organ under physiological conditions, in the present study, we employed this approach to investigate the interplay between inflammation and CFTR modulator efficacy in a pancreatic epithelial context, without aiming to replicate bacterial infection per se.

We investigated the effects of the CFTR modulators VX770, VX661, and VX445, administered alone or in combination, on pancreatic epithelial cell physiology, using CFPAC-1 cells, a human pancreatic ductal cell line that endogenously expresses the F508del mutation [33]. CFPAC-1 cells retain key morphological and functional characteristics of the pancreatic ductal epithelium and have already been used as an in vitro model to study CF-related pancreatic dysfunction [34,35,36,37]. To promote epithelial polarization and differentiation in a manner that mimics the physiological pancreatic microenvironment, the cells were cultured at either liquid-liquid interface (LLI) or air-liquid interface (ALI). We used a multidisciplinary approach, combining measurements of transepithelial electrical conductance and fluid reabsorption, as well as assessment of apical surface fluid (ASF) pH and viscoelastic properties, to comprehensively evaluate the impact of CFTR modulators on pancreatic epithelial function under both basal and pro-inflammatory conditions. Our results demonstrate that, in CFPAC-1 epithelial layers, VX661 and VX445, particularly when used in combination with VX770, enhance CFTR-dependent ion and fluid transport, increase ASF pH, and reduce mucus microviscosity. Notably, the efficacy of these modulators was maintained, and in some cases, even slightly enhanced, under pro-inflammatory conditions, supporting their therapeutic potential in addressing pancreatic dysfunction in CF. Notably, the CFPAC-1 cell line provides a valuable and widely accepted model for investigating CFTR-related defects in a pancreatic context; however, its tumor origin and potential differentiation-related limitations should be taken into account when extrapolating findings to native ductal epithelium. Complementary studies using primary pancreatic cells will be important to validate the physiological relevance of the obtained results.

## 2. Materials and Methods

### 2.1. Chemicals

Ivacaftor (VX770, N-(2,4-ditert-butyl-5-hydroxyphenyl)-4-oxo-1H-quinoline-3-carboxamide), tezacaftor (VX661, 1-(2,2-difluoro-1,3-benzodioxol-5-yl)-N-[1-[(2R)-2,3-dihydroxypropyl]-6-fluoro-2-(2-hydroxy-1,1-dimethylethyl)-1H-indol-5-yl]-cyclopropanecarboxamide), and elexacaftor (VX445, (S)-N-((1,3-dimethyl-1H-pyrazol-4-yl)sulfonyl)-6-(3-(3,3,3-trifluoro-2,2-dimethylpropoxy)-1H-pyrazol-1-yl)-2-(2,2,4-trimethylpyrrolidin-1-yl)nicotinamide) were purchased from Selleck Chemicals (Munich, Germany). The CFTR inhibitor PPQ102 (12,14-dimethyl-9-(5-methylfuran-2-yl)-17-phenyl 1,8,12,14tetrazatetracyclo [8.7.0.02,7.011,16]heptadeca-2,4,6,10,16-pentaene-13,15-dione) and forskolin (Fsk, 3R,4aR,5S,6S,6aS,10S,10aR,10bS-3-ethenyl-6,10,10b-trihydroxy-3,4a,7,7,10a-pentamethyl-1-oxo-5,6,6a,8,9,10-hexahydro-2H-benzo[f]chromen-5-yl] acetate) were obtained from Merck (Milan, Italy). Unless otherwise specified, all other reagents and chemicals were obtained from Merck (Milan, Italy).

### 2.2. Cell Culture

CFPAC-1 cells, immortalized pancreatic ductal epithelial cells expressing the F508del mutation in the CFTR gene, originally obtained from a patient with CF [33], were seeded at a high density (2.5 × 10^5^ cells per well) onto permeable polycarbonate Snapwell supports (0.4 μm pore size, 1.12 cm diameter; Corning, New York, NY, USA), which had been pre-coated with rat tail collagen type I. The cells were cultured under liquid–liquid interface (LLI) conditions in DMEM supplemented with 10% fetal bovine serum, 100 U/mL penicillin-streptomycin, and 2 mM L-glutamine. The medium was replaced every 48 h until a stable, confluent epithelial layer was established, which typically took 7–10 days. Epithelial integrity was verified by monitoring transepithelial electrical resistance (TEER) using a voltohmmeter (EVOM2, World Precision Instruments, Sarasota, FL, USA).

For selected experiments (e.g., fluid reabsorption and MPT assays), the cells were transitioned to air–liquid interface (ALI) conditions to promote apical surface exposure and mucus secretion. For ALI culture, the medium on the apical surface was removed, and epithelial preparations were maintained for a further 5–7 days with only the basolateral medium. Morphological integrity, mucus presence, and TEER stability were verified prior to experimental procedures.

For the experiments aimed at evaluating the mucin secretion, HEK-293 (human Embryonic Kidney) and Calu-3 (human bronchial epithelial) cells were grown in Dulbecco’s modified Eagle’s medium (DMEM) or DMEM:F-12, both supplemented with 10% fetal bovine serum, 100 UI/mL penicillin, 100 μg/mL streptomycin, 20 mM L-glutamine, and 1% *v*/*v* non-essential amino acids, respectively. The Calu-3 cells were seeded onto permeable polycarbonate Snapwell supports and grown under the ALI condition, as previously described.

### 2.3. Pharmacological Treatments and Inflammatory Stimulation

To assess functional rescue of F508del CFTR, mature CFPAC-1 epithelial preparations were treated with CFTR modulators. Tezacaftor (VX661, 5 μM), elexacaftor (VX445, 5 μM), or their combination (VX661, 5 μM + VX445, 5 μM) were dissolved in 0.1% (*v*/*v*) DMSO and applied to the basolateral compartment for 24 h to allow the correctors sufficient time to promote proper folding, trafficking, and apical localization of the F508del CFTR protein. When combined, VX661 and VX445 were applied simultaneously at 5 μM each, for a total final concentration of 10 μM.

DMSO (0.1% *v*/*v*) alone was used as a vehicle control.

To induce an inflammatory response, lipopolysaccharide (LPS) from *Escherichia coli* O111:B4 (Merck) was added to the basolateral medium at a final concentration of 10 μg/mL and maintained for 24 h [38]. LPS exposure was continued during CFTR corrector treatment [39,40,41,42,43,44].

Ten minutes prior to functional measurements, cells (except the DMSO control) were acutely stimulated with forskolin (Fsk, 20 μM), applied to both apical and basolateral compartments to activate cAMP-dependent CFTR signaling. Ivacaftor (VX770, 1 μM) was co-applied with FSK to assess its potentiating effect on the gating function of CFTR channels already localized at the apical membrane.

In selected experiments, CFTR inhibition was achieved by applying PPQ102 (30 μM) to both apical and basolateral compartments 10 min after Fsk + VX770 stimulation.

### 2.4. Transepithelial Electrical Resistance (TEER) Measurements

Transepithelial electrical resistance (TEER) was measured using the EVOM2 voltohmmeter (World precision Instrument, Sarasota, FL, USA) with STX2 chopstick electrodes. Measurements were performed at 37 °C, and data were normalized to the surface area of the insert (1.12 cm^2^). TEER values were converted to transepithelial conductance (TEC, in μS/cm^2^) using the formula TEC (G) = 1/TEER. Baseline values (G_pre) were recorded immediately before stimulation. Final values (G_post) were obtained 10 min after Fsk + VX770 treatment. In conditions with PPQ102, a third measurement was recorded 10 min after inhibitor addition. CFTR-dependent ion transport was evaluated by measuring the change in transepithelial conductance (ΔG = G_post − G_pre) before and after stimulation. All experiments were performed in replicate (n ≥ 8 per condition), and results were reported as mean ± standard error of the mean (SEM).

### 2.5. Measurement of Fluid Absorption (Jw) and Total Protein Content of the Apical Surface Fluid (ASF)

To assess epithelial fluid absorption, 150 μL of isotonic buffer solution (NaCl 150 mM, HEPES 10 mM, pH 7.4) was applied to the apical surface of ALI-cultured epithelia, either with or without LPS-induced pro-inflammatory stimulation. After 24 h of incubation at 37 °C and 5% CO_2_ in the presence of CFTR modulators, the residual apical fluid was collected and weighed in pre-weighed tubes. The final volume was calculated gravimetrically, assuming a density of 1 g/mL. Net fluid absorption rate (Jw) was calculated as follows:

Jw = (Vi − Vf)/(A × t),(1)

where Vi = 150 μL, Vf = final volume, A = 0.33 cm^2^, and t = 24 h.

Cell-free blank inserts were used to control for evaporation and pipetting variability. Results were expressed in μL·h^−^^1^·cm^−^^2^ as mean ± SEM (n ≥ 6). The method was adapted from previous studies employing gravimetric quantification of fluid absorption [45,46,47].

Total protein concentration in the collected apical surface fluid was measured with Bradford assay at 595 nm (Jenway 6705 UV/Vis, Cole-Parmer, Vernon Hills, IL, USA) using BSA as standard. Results were expressed in μg/μL as mean ± SEM (n ≥ 4). Samples not immediately analyzed were stored at −80 °C for up to three weeks.

### 2.6. pH Measurement in Apical Surface Fluid (ASF)

After 24 h of treatment with CFTR modulators, the ASF was collected from ALI-cultured CFPAC-1 epithelia and its pH was measured using a microelectrode (SevenCompact, Mettler Toledo, Novate Milanese, Italy). To ensure pH stability, measurements were performed immediately after collection, and the time between sampling and analysis did not exceed 2 min.

The instrument was calibrated using standard buffer solutions at pH 7.00 and pH 4.00. Each condition was tested in at least 6 replicates, and results are reported as mean ± SEM.

### 2.7. Western Blot and Dot Blot Analyses

Western blot analysis was performed on whole cell lysates and basolateral fluids of CFPAC-1 epithelial preparations in order to examine the expression of the F508del CFTR and the pro-inflammatory cytokines IL-6, IL-8, and IL-1β, respectively. Briefly, the CFPAC-1 layers were rinsed with PBS and lysed in RIPA buffer containing protease and phosphatase inhibitors. To detect secreted cytokines, the basolateral media were concentrated using Amicon Ultra-0.5 mL centrifugal filter units with a 3 kDa cut-off (MWCO) at 14,000× *g* for 20 min at 4 °C.

Protein concentration (μg/μL) in both whole cell lysates and concentrated basolateral fluid was determined using the Bradford assay (Bio-Rad, Hercules, CA, USA), according to the manufacturer’s protocol. Aliquots corresponding to 30 μg of total protein were separated on 12% SDS-PAGE gels and transferred to PVDF membranes (MilliporeSigma, Burlington, MA, USA). After blocking in 5% milk in PBS containing 0.1% Tween-20 (PBS-T), membranes were incubated overnight at 4 °C with primary antibodies: CFTR (mouse monoclonal, 1:200, clone MM13-4; MilliporeSigma), IL-6 (rabbit polyclonal, 1:100; Thermo Fisher Scientific, Waltham, MA, USA), IL-8 (rabbit monoclonal, 1:100; Abcam, Cambridge, UK), or IL-1β (rabbit polyclonal, 1:100; Thermo Fisher Scientific). After washing, HRP-conjugated secondary antibodies (goat anti-mouse or anti-rabbit, 1:2000; Santa Cruz Biotechnology, Dallas, TX, USA) were applied for 1 h. Detection was performed using Amersham ECL Plus (GE Healthcare Europe GmbH, Milan, Italy), and signals were visualized with Amersham Hyperfilm ECL. For secreted cytokines, Coomassie Blue staining (R250, Biorad) of the SDS-PAGE gels was performed prior to the immunodetection procedure to verify uniform protein loading and to enable semi-quantitative comparisons. For CFTR expression, membranes were stripped (62.5 mM Tris–HCl pH 6.8, 10% SDS, 1% β-mercaptoethanol, 55 °C for 30 min) and re-probed with anti-actin antibody (polyclonal, 1:2000; Merck) to ensure equal protein loading. For CFTR quantification, band intensities were normalised to actin from the same lane on the re-probed membranes. For secreted cytokines, the band intensities were normalized to the corresponding Coomassie-stained lanes. Densitometric analysis was performed using ImageJ software (version 1.53t, NIH, Bethesda, MD, USA) [48]. All values were expressed relative to the Fsk-treated LPS-unstimulated sample, which was set to 1. At least four biological replicates were analyzed per condition.

As part of the phenotypic characterization of the CFPAC-1 model grown under air–liquid interface (ALI) conditions, dot blot analysis was performed to assess mucin secretion. The protein concentration of ASF was measured using the Bradford assay. Control untreated ASF was concentrated using Amicon Ultra-0.5 mL centrifugal filter units, and an aliquot of 10 µL (approximately 20 µg of total protein) was spotted directly onto nitrocellulose membranes (Amersham Protran, GE Healthcare). The membranes were air-dried at room temperature and incubated overnight at 4 °C with primary antibodies against MUC1 (mouse monoclonal, 1:500, Thermo Fisher Scientific, Waltham, MA, USA) and MUC5B (rabbit polyclonal, 1:200, Thermo Fisher Scientific) in PBS-T. After washing with PBS-T, the membranes were incubated with HRP-conjugated anti-mouse or anti-rabbit secondary antibodies (1:2500) for 1 h at room temperature. Membranes were processed using standard immunodetection procedures. To verify the specificity of the antibodies, dot blot assay was also performed using 10 µL of concentrated (2 μg/μL) apical surface liquid (ASL) collected from Calu-3 cells, cultured under the ALI condition, while 10 µL of concentrated (2 μg/μL) culture medium collected from HEK-293 cells cultured under standard conditions was used as a negative control due to the lack of mucus production in these cells.

### 2.8. Multiple Particle Tracking (MPT) Analysis

To evaluate the microrheological properties of the apical surface fluid (ASF), the MPT assay was performed on ASF samples collected from ALI-cultured CFPAC-1 cell layers [45,46,47,49,50]. A 25 μL sample of ASF was mixed with 1 μL of yellow-green fluorescent carboxylated polystyrene beads (200 nm diameter, λ_exc = 488 nm; λ_em = 505–515 nm, Life Technologies, Monza, Italy). A total of 8 μL of the mixture was sealed between glass coverslips to prevent evaporation and equilibrated for 20 min at room temperature. All MPT measurements were performed at room temperature (approximately 22–24 °C), with consistent conditions applied to all samples to ensure comparability across treatments. The beads were then imaged at mid-height using a 60× oil-immersion objective (NA 1.42) connected to a CCD camera. Videos (1280 × 960 pixels) were acquired at 5 frames/s. Brownian trajectories were analyzed using the Multitracker plug-in of the ImageJ software [48], tracking ~400 beads from 4 to 8 fields per sample. The movement of the fluorescent beads within the ASF in a given time interval, τ, is described by its mean squared displacement, <msd>, from which it is possible to compute the diffusion coefficient D_0_, according to the following equation:

〈msd(τ)〉 = 4 D_0_τ^α^, (2)

where τ and α represent the time interval and the elastic contribution of the fluid (0 < α ≤ 1), respectively.

The microviscosity (η) was calculated via the Stokes–Einstein equation:

η = K_B_ T/6π D_0_ r, (3)

where K_B_ is Boltzmann’s constant, T is absolute temperature, and r is the bead radius. Microviscosity values were expressed in centipoise (cP), where 1 cP = 0.001 Pa·s. This unit reflects a model-derived estimation of fluid microviscosity based on the thermal diffusion behavior of the probe particles.

### 2.9. Statistics

Initial ANOVA testing was performed to assess variability within and between groups. Once homogeneity had been confirmed, data from 4–12 replicates per condition were pooled. Each experiment was independently repeated using at least three distinct cell culture passages to ensure biological reproducibility. Sample sizes were chosen to provide sufficient resolution for detecting treatment-dependent differences while maintaining technical feasibility within the constraints of in vitro experimentation. The results are presented as mean ± standard error of the mean (SEM). All statistical and MPT analyses were performed using IgorPro 9 software (WaveMetrics, Lake Oswego, OR, USA). Multiple comparisons were performed using the Kruskal–Wallis non-parametric ANOVA, followed by Dunn’s post hoc test. A value of *p* < 0.05 was considered statistically significant.

## 3. Results

### 3.1. CFTR Modulators Enhance Transepithelial Conductance (TEC) in CFPAC-1 Epithelial Preparations

To evaluate the ability of CFTR modulators to restore CFTR functional expression in CFPAC-1 epithelial preparations, transepithelial electrical conductance (TEC) was measured before and after treatment with the potentiator VX770 and the correctors VX661 and VX445, administered either individually or in combination. With the exception of the DMSO control condition, the cAMP activator forskolin (Fsk) was included in all other experimental conditions. The specificity of the CFTR-mediated response was verified using the CFTR inhibitor PPQ102. Pro-inflammatory conditions were modelled by preconditioning the epithelial preparations with lipopolysaccharide (LPS).

CFPAC-1 cells cultured on permeable supports formed confluent layers, exhibiting a baseline TEC of 3033 ± 29.51 μS/cm^2^ (n = 72). This corresponded to a transepithelial electrical resistance (TEER) of approximately 330 ± 3.2 Ω·cm^2^, a value consistent with those reported by other research groups under comparable experimental conditions [35].

**Figure 1:**
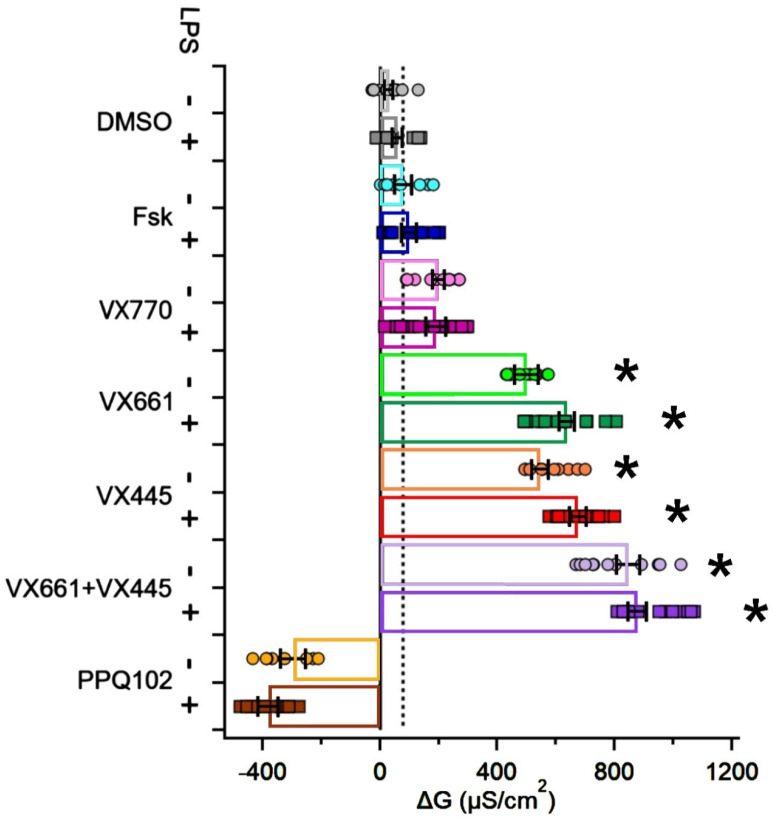
The effect of CFTR modulators on transepithelial electrical conductance (ΔTEC) in CFPAC−1 epithelial preparations, both with and without LPS stimulation. The Bar graph shows the changes in TEC (ΔG, μS/cm^2^) measured in CFPAC−1 epithelial preparations after treatment with vehicle (0.1% DMSO), forskolin (Fsk, 20 μM), VX770 (1 μM), VX661 (5 μM), VX445 (5 μM), VX661 (5 μM) + VX445 (5 μM) or the CFTR inhibitor PPQ102 (30 μM). Each condition was tested in the absence (−) or presence (+) of lipopolysaccharide preconditioning (LPS, 10 μg/mL, 24 h). Bars represent the mean ± standard error of the mean (SEM) of at least n = 8 independent experiments. Circles (−LPS) and squares (+LPS) indicate individual data points. Statistical comparisons were performed using the Kruskal–Wallis ANOVA followed by Dunn’s post hoc test. Comparisons were made versus the Fsk−treated, LPS-unstimulated group, whose mean value is represented by the black dashed line. Asterisks (*) indicate statistically significant differences (* *p* < 0.05).

In the absence of LPS, Fsk alone induced a modest increase in conductance (ΔTEC = 78.19 ± 27.25 μS/cm^2^, n = 8) with respect to DMSO (ΔTEC = 30.68 ± 13.45 μS/cm^2^, n = 12). Similarly, treatment with Fsk + VX770 (hereafter referred to as VX770) produced a comparable, though non-significant, increase in conductance (ΔTEC = 199.42 ± 20.40 μS/cm^2^, n = 12). In contrast, dual combinations of CFTR potentiator with either VX661 or VX445 (hereafter referred to as VX661 and VX445, respectively) led to significantly greater increases in TEC (ΔTEC = 498.64 ± 40.97 μS/cm^2^, n =11 and ΔTEC = 544.36 ± 28.26 μS/cm^2^, n = 10, respectively). The triple combination VX661 + VX445 + VX770 (hereafter referred to as VX661 + VX445) produced the greatest increase in epithelial conductance (ΔTEC = 844.42 ± 39.4 μS/cm^2^, n = 12). As expected, inhibition of CFTR function with PPQ102 significantly decreased TEC (ΔTEC = −298.05 ± 42.12 μS/cm^2^, n = 8). Figure 1 and Table S1 summarize the obtained results, with Table S1 reporting the statistical comparisons performed between each CFTR modulator treatment and the DMSO-treated, LPS-unstimulated control group, together with the corresponding *p*-values.

Under the pro-inflammatory conditions induced by LPS, modulator responses were generally preserved or modestly enhanced. Forskolin alone increased conductance to a similar extent (ΔTEC = 98.23 ± 25.16 μS/cm^2^, n = 12) as VX770 (ΔTEC = 188.41 ± 34.65 μS/cm^2^, n = 12), with no statistically significant difference compared with the non-inflamed forskolin response. By contrast, VX661 (ΔTEC = 635.21 ± 26.2 μS/cm^2^, n = 12), VX445 (ΔTEC = 673.59 ± 27.68 μS/cm^2^, n = 12), and VX661 + VX445 (ΔTEC = 877.87 ± 30.43 μS/cm^2^, n = 12) promoted significantly enhanced conductance values compared with forskolin alone under non-inflamed conditions. Notably, PPQ102 retained its inhibitory effect under pro-inflammatory stress, leading to a marked decrease in conductance (−383.24 ± 33.37 μS/cm^2^, n = 12, Figure 1 and Table S1).

### 3.2. CFTR Modulators Reduce Apical Surface Fluid Reabsorption in CFPAC-1 Monolayers

**Figure 2:**
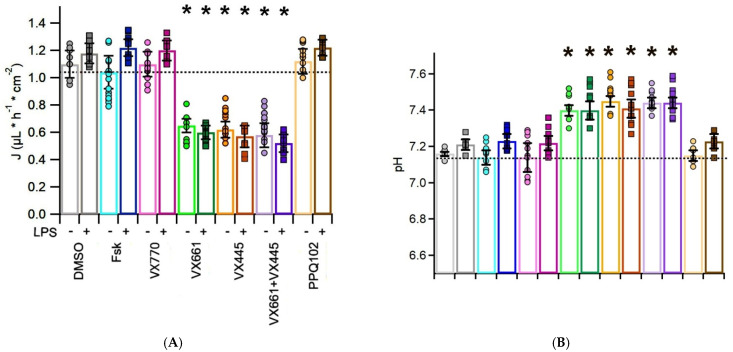
CFTR modulators improve fluid reabsorption and alkalinize the pH of the apical surface fluid (ASF) of CFPAC−1 epithelial layers. Bar graphs showing (**A**) Fluid reabsorption rate (J) and (**B**) the pH of the ASF pH in CFPAC−1 epithelial layers treated with vehicle (0.1% DMSO), Fsk (20 μM), VX770 (1 μM), VX661 (5 μM), VX445 (5 μM), their combination (5 μM VX661 + 5 μM VX445), or the CFTR inhibitor PPQ102 (30 μM). Experiments were conducted in the absence (−) or presence (+) of lipopolysaccharide (LPS, 10 μg/mL, 24 h). Fluid reabsorption was measured gravimetrically while the pH was assessed using a microelectrode. Bars represent the mean ± standard error of the mean (SEM) of at least n = 6 replicates. Circles (−LPS) and squares (+LPS) indicate individual data points. Statistical comparisons were performed using the Kruskal–Wallis ANOVA followed by Dunn’s post hoc test. Comparisons were made versus the Fsk−treated, LPS−unstimulated group, whose mean value is represented by the black dashed line. Asterisks (*) indicate statistical significance (* *p* < 0.05).

To evaluate the effects of CFTR modulators on apical fluid dynamics, the rate of apical fluid reabsorption (J, expressed in μL·h^−1^·cm^−2^) was measured in CFPAC-1 epithelial preparations following treatment with CFTR modulators, in both the presence and absence of the pro-inflammatory stimulus LPS. In this experimental model, CFTR activation leads to chloride and fluid secretion toward the apical compartment, resulting in a reduction of net fluid reabsorption; therefore, lower J values indicate improved CFTR function. The results are summarized in Figure 2A and Table S2, with Table S2 also providing the statistical comparisons between each CFTR modulator treatment and the DMSO-treated, LPS-unstimulated control group, together with the associated *p*-values.

Treatment with VX770 yielded a mean absorption rate of 1.10 ± 0.09 μL·h^−1^·cm^−2^ (n = 10), which was comparable to the rates observed with Fsk (1.04 ± 0.12 μL·h^−1^·cm^−2^, n = 8) and DMSO (1.1 ± 0.10 μL·h^−1^·cm^−2^, n = 9). This indicates that VX770 alone did not significantly stimulate CFTR-mediated reabsorption in CFPAC-1 cells, consistent with the absence of corrected CFTR at the plasma membrane. In contrast, the addition of CFTR correctors significantly reduced fluid reabsorption. Cells treated with VX661 or VX445 displayed markedly lower J values (0.65 ± 0.05 μL·h^−1^·cm^−2^, n = 8 and 0.62 ± 0.06 μL·h^−1^·cm^−2^, n = 10, respectively), while the combination VX445 + VX661 had the most pronounced reduction in fluid reabsorption (0.58 ± 0.09 μL·h^−1^·cm^−2^, n = 12), reflecting enhanced CFTR activity.

In the presence of LPS, the baseline absorption (Fsk + LPS) increased to 1.22 ± 0.06 μL·h^−1^·cm^−2^ (n = 10), suggesting that the inflammatory environment may alter epithelial transport dynamics subtly, possibly favoring increased apical fluid accumulation. Similarly, the response to DMSO and VX770 was not improved by LPS, with J values of 1.18 ± 0.07 μL·h^−1^·cm^−2^ (n = 10) and 1.20 ± 0.07 μL·h^−1^·cm^−2^ (n = 10), respectively.

Notably, LPS preconditioning resulted in significant reductions in J in all corrector-treated conditions, reaching 0.60 ± 0.05 μL·h^−1^·cm^−2^ (n = 12) with VX661, 0.57 ± 0.08 μL·h^−1^·cm^−2^ (n = 12) with VX445, and 0.52 ± 0.06 μL·h^−1^·cm^−2^ (n = 12) with VX661 + VX445. Finally, the CFTR inhibitor PPQ102 reversed the fluid absorption effect when administered after Fsk + VX770 stimulation, both in the absence and presence of LPS. Indeed, it yielded J values comparable to those of Fsk-treated, LPS unstimulated samples: 1.12 ± 0.09 μL·h^−1^·cm^−2^ (n = 6) and 1.22 ± 0.06 μL·h^−1^·cm^−2^ (n = 9), respectively.

### 3.3. CFTR Modulators Increase the pH of the Apical Surface Fluid in CFPAC-1 Cells, with Additive Effects in the Presence of LPS

To investigate ion transport and bicarbonate secretion in CFPAC-1 cell layers, the ASF was carefully collected and its pH recorded immediately using a microelectrode [46,47].

In the absence of LPS, the pH of DMSO-treated epithelia was 7.16 ± 0.01 (n = 8). Fsk and VX770 treatment did not significantly modify the pH (7.14 ± 0.04, n = 8 and 7.14 ± 0.08, n = 12, respectively). Conversely, treatment with VX661 and VX445 increased the ASL pH to 7.4 ± 0.03 (n = 8) and 7.45 ± 0.04 (n = 12), respectively. The combination VX661 + VX445 further increased the pH to 7.44 ± 0.03 (n = 11). PPQ102 slightly reduced the ASL pH to 7.15 ± 0.03 (n = 7) (see Figure 2B and Table S3).

Following LPS preconditioning, the pH of the DMSO-treated fluid samples increased modestly to 7.21 ± 0.03 (n = 8) compared with the unstimulated LPS condition. Fsk and VX770 produced slight, non-significant increases in pH (7.23 ± 0.04, n = 12 and 7.22 ± 0.04, n = 11, respectively). VX661 (7.4 ± 0.05, n = 8), VX445 (7.41 ± 0.05, n = 12), and VX661 + VX445 (7.44 ± 0.03, n = 12) significantly increased the alkalinity of the ASF. Finally, under LPS stimulation, ASF samples treated with PPQ102 displayed a slightly higher pH (7.23 ± 0.04, n = 6) than under non-inflamed conditions (7.15 ± 0.03, n = 7), which may indicate LPS-induced modulation of pH-regulating pathways independently of CFTR. The results are summarized in Figure 2B and Table S3, which also reports the statistical comparisons performed between each CFTR modulator treatment and the DMSO-treated, LPS-unstimulated control group, together with the corresponding *p*-values.

Overall, these findings demonstrate that CFTR modulators enhance bicarbonate secretion and make the ASF more alkaline in F508del CFTR-expressing CFPAC-1 epithelial layers, and that a pro-inflammatory stimulus, such as LPS, further enhances this response.

### 3.4. CFTR Modulators Do Not Affect Total Protein Concentration in the Apical Surface Fluid (ASF) of CF-PAC1 Epithelial Layers

The total protein concentration in the ASF was quantified using the Bradford assay and expressed in μg/μL. To avoid procedures requiring destructive sampling, which would compromise the integrity of ALI-cultured epithelial layers, normalization of the protein content to cell number or surface area was not performed. Therefore, protein values were reported relative to apical fluid volume only. In the absence of LPS, apical fluid collected from Fsk-treated CFPAC-1 layers displayed an average protein concentration of 1.36 ± 20.21 μg/μL (n = 5). No significant differences were observed among the different treatment groups, indicating that CFTR modulators did not affect overall protein secretion under basal conditions (see Table 1).

Following LPS preconditioning, a significant increase in apical protein concentration was observed in all experimental groups compared to the Fsk-treated, LPS-unstimulated group. Specifically, the average protein concentration in the ASF of Fsk-treated layers exposed to LPS was 1.68 ± 22.53 μg/μL (n = 6). However, no significant differences were observed among the different treatments within the LPS-stimulated conditions, suggesting that the modulators do not differentially impact protein secretion during inflammation.

### 3.5. CFTR Modulators Enhance Endogenous F508del CFTR Levels in CFPAC-1 Cells but Do Not Reduce Pro−Inflammatory Cytokine Expression

Western blot analysis was performed to evaluate the expression levels of endogenous F508del CFTR protein in CFPAC-1 whole cell lysates from CFPAC-1 epithelial preparations, as well as of IL-6, IL-8 and IL-1β, pro-inflammatory cytokines secreted into the basolateral medium [28,51,52,53,54,55].

**Figure 3:**
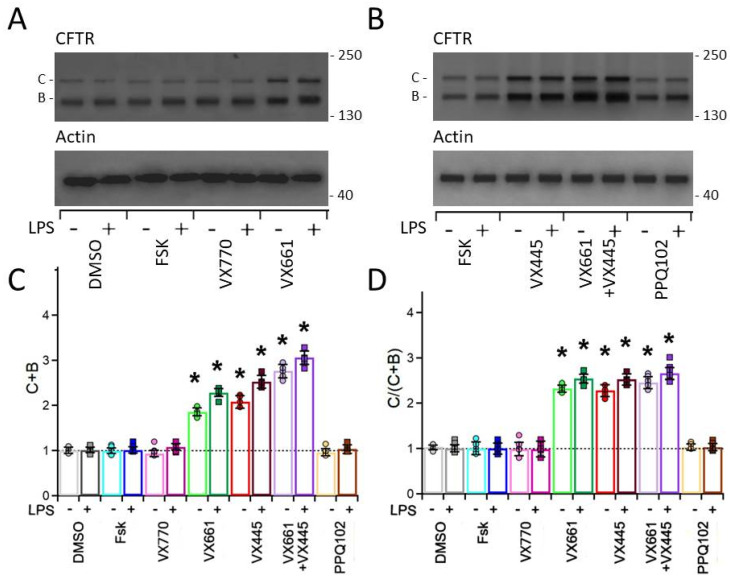
Detection and quantification of F508del CFTR protein expression in CFPAC−1 epithelial layers by Western blot analysis. (**A**,**B**) Representative Western blots showing F508del CFTR expression in whole-cell lysates from CFPAC-1 epithelial layers treated with vehicle control (0.1% DMSO), forskolin (Fsk, 20 μM), VX770 (1 μM), VX661 (5 μM), VX445 (5 μM), VX661 (5 μM) + VX445 (5 μM) combination, and PPQ102 (30 μM). The upper panels show the F508del CFTR protein detected using the monoclonal antibody MM13-4, which is directed against the CFTR N-terminus. The lower panels illustrate the immunoblotting of actin, which was used as a loading control. Molecular weight markers (in kDa) are indicated to the left of each blot. The positions of the immature, core-glycosylated ER-resident form (band B) and the mature, fully glycosylated form (band C) of CFTR are also indicated on the right of each blot. (**C**,**D**) Bar graphs showing the densitometric quantification of total CFTR expression (the sum of bands B + C) and the CFTR maturation rate (the ratio of the mature band C to the total CFTR bands C/(C + B)). Band intensities were normalised to actin and expressed relative to Fsk-treated, LPS-unstimulated samples, whose mean was set at 1 (black dashed line). Bars represent the mean ± SEM from at least four independent experiments. Individual data points are indicated by circles (−LPS preconditioning) and squares (+LPS preconditioning). Statistical comparisons were performed using the Kruskal–Wallis non-parametric analysis of variance followed by Dunn’s post hoc test. Comparisons were made versus the Fsk-treated, LPS-unstimulated group, whose mean value is represented by the black dashed line. Asterisks (*) indicate statistically significant differences (* *p* < 0.05).

The F508del CFTR isoform was detected by the monoclonal antibody MM13-4, directed against the CFTR N-terminus, as two electrophoretic bands: band B (~160 kDa), corresponding to the immature, core-glycosylated form retained in the endoplasmic reticulum (ER), and band C (~180 kDa), corresponding to the mature, fully glycosylated isoform processed through the Golgi apparatus. As expected, CFPAC-1 cells predominantly expressed the immature CFTR band B, consistent with the severe folding and trafficking defect associated with the F508del mutation (see first lane of the upper panel in Figure 3A,B). Densitometric analysis revealed that treatment with Fsk or VX770 did not significantly modify the total CFTR expression (sum of bands B + C) or maturation rate (ratio of band C to the sum of bands B + C). In contrast, treatment with VX661 and VX445, and particularly their combination (VX661 + VX445), significantly increased both the total expression and maturation rate of F508del CFTR (see Figure 3C,D). Notably, the effect of correctors also included a marked increase in the immature CFTR band B, suggesting that treatment may enhance overall CFTR protein expression, potentially through effects on protein synthesis and/or stability (see Figure 3A,B).

Although the effects of CFTR modulators in LPS-preconditioned samples did not lead to a statistically significant increase in CFTR total protein expression or maturation rate compared with LPS non-preconditioned samples, the modulators remained effective under inflammatory conditions, indicating that their corrective activity is preserved in a pro-inflammatory epithelial environment. Specifically, under LPS preconditioning, Fsk or VX770 alone had no effect on CFTR expression or processing. Treatments with VX661 and VX445 produced comparable levels of CFTR rescue, whereas their combination exhibited the most robust effect, increasing total CFTR expression by approximately threefold compared with Fsk-treated, non-LPS-stimulated samples (see Figure 3C,D). Quantitative data for CFTR total expression and maturation rate, normalized to actin (used as a housekeeping protein), and referred to the Fsk-treated LPS-unstimulated whole cell lysates (set to 1), are reported in Table S4, which also provides the statistical comparisons between each CFTR modulator treatment and the DMSO-treated, LPS-unstimulated control group, together with the associated *p*-values.

**Figure 4:**
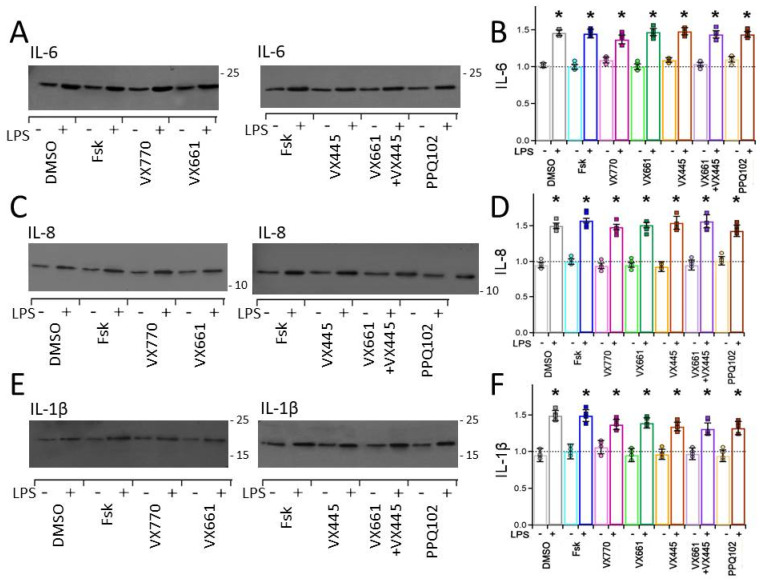
Detection of interleukins secreted by ALI−cultured CFPAC−1 epithelial layers. Representative Western blots demonstrating the expression of (**A**) IL−6, (**C**) IL−8, and (**E**) IL−1β in concentrated basolateral medium from CFPAC−1 epithelial layers that were treated with vehicle (0.1% DMSO), forskolin (Fsk, 20 μM), VX770 (1 μM), VX661 (5 μM), VX445 (5 μM), VX661 (5 μM)+ VX445 (5 μM) combination, and PPQ102 (30 μM). Molecular weight markers (in kDa) are indicated to the left of each blot. Densitometric quantification of (**B**) IL−6, (**D**) IL−8, and IL−1β (**F**) expression levels. Band intensities were normalised to the Coomassie signal of the corresponding SDS−PAGE gels and expressed relative to Fsk−treated LPS−unstimulated samples, whose mean was set at 1 and indicated by the black dashed line. Bars represent the mean ± SEM from at least four independent experiments. Individual data points are shown as circles for −LPS preconditioning and squares for +LPS preconditioning. Statistical analysis was assessed using Kruskal–Wallis non−parametric analysis of variance followed by Dunn’s post hoc test. Comparisons were made versus the Fsk−treated, LPS−unstimulated group. Asterisks (*) indicate statistically significant differences (* *p* < 0.05).

Under basal conditions (DMSO and Fsk treatments), the levels of IL-6, IL-8, and IL-1β detected in the concentrated basolateral media were relatively low, indicating minimal spontaneous inflammatory activity. Cytokine expression in samples treated with CFTR modulators (VX770, VX661, and VX445, either individually or in combination) did not differ significantly from those treated with DMSO and Fsk (see Figure 4A–F). Following stimulation with LPS, CFPAC-1 epithelial layers treated with DMSO and Fsk exhibited significantly higher levels of IL-6, IL-8, and IL-1β secreted in the basolateral medium compared with non-stimulated controls (see Figure 4A–F). Notably, cytokine levels remained elevated in samples treated with CFTR modulators (VX770, VX661, VX445, either individually or in combination) and were comparable to those observed in samples treated with DMSO- and Fsk- samples stimulated with LPS (see Figure 4A–F). These findings confirm that LPS effectively induces a pro-inflammatory response in CFPAC-1 epithelia. Furthermore, under these specific conditions of pro-inflammatory stimulation (10 μg/mL LPS, 24 h), our data demonstrate that CFTR modulators did not exert detectable anti-inflammatory effects on IL-6, IL-8, and IL-1β cytokine production in CFPAC-1 epithelial layers. Semi-quantitative data for interleukins, normalized to the Coomassie-stained gels of concentrated basolateral medium from CFPAC-1 cultures and expressed relative to the Fsk-treated, LPS-unstimulated whole-cell lysates (set to 1), are reported in Table S5. The table also includes the statistical comparisons between each CFTR modulator treatment and the DMSO-treated, LPS-unstimulated control group, together with the corresponding *p*-values. A representative Coomassie Blue staining of the gels used for protein normalization is shown in Figure S1, illustrating the quality and uniformity of protein loading across the samples.

### 3.6. CFTR Modulators Promote Mucin Secretion in the Apical Surface Fluid (ASF) of CFPAC-1 Epithelial Preparations

To verify the secretory phenotype of CFPAC-1 epithelial layers grown under ALI conditions, dot blot analyses were performed on concentrated ASF collected from DMSO-treated control cultures. Dot blot analysis revealed the presence of mucin MUC1 but not of MUC5B in the ASF collected from CFPAC-1 epithelia cultured under the ALI conditions (see the first spots in the upper and lower blots of Figure S2), confirming that CFPAC-1 cells can produce and secrete organ-specific mucins at the apical surface under ALI conditions [56,57,58,59,60]. To validate the reliability of mucin detection, parallel dot blot analyses were performed using the apical fluid collected from ALI-cultured Calu-3 bronchial epithelial cells, a well-established mucus-producing cell line [61,62]. As expected, strong signals for MUC1 (second spot in the upper blot of Figure S2, [63,64,65]) and MUC5B (second spot in the lower blot of Figure S2) were detected in the ASL samples from Calu-3 cells, confirming the specificity of the antibodies used and the reliability of the assay [63,64,66]. Conversely, no mucin signals were detected in the culture medium collected from HEK-293 cells (see the third spot of the upper and lower blots of Figure S2), which served as a negative control due to their non-mucus-producing phenotype. This further validated the specificity of the mucin detection protocol.

### 3.7. CFTR Modulators Decrease the Viscosity of the Apical Surface Fluid (ASF) in CFPAC-1 Epithelial Layers

**Figure 5:**
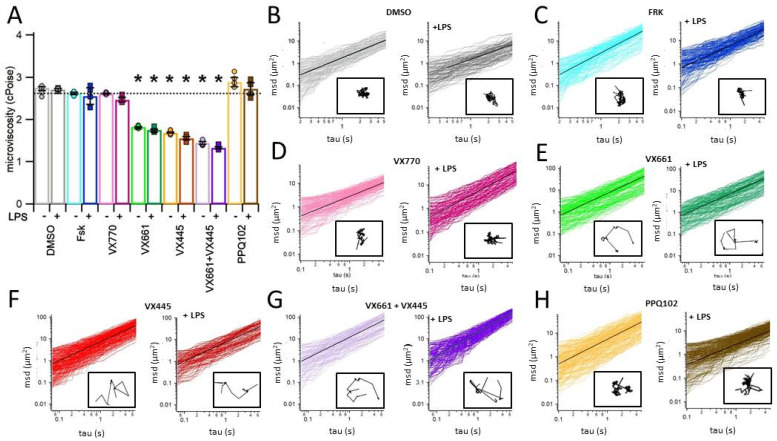
Microviscosity analysis of the apical surface fluid (ASF) secreted by ALI−cultured CFPAC−1 epithelial layers, as measured using multiple particle tracking (MPT). (**A**) Bar graph showing the microviscosity of ASF collected from the apical surface of CFPAC−1 layers treated with vehicle (0.1% DMSO), forskolin (Fsk, 20 μM), the CFTR potentiator VX770 (1 μM), correctors VX661 (5 μM) and VX445 (5 μM), their combination (VX661, 5 μM + VX445, 5 μM), or the CFTR inhibitor PPQ102 (30 μM). Treatments were performed in the absence (−LPS) or presence (+LPS) of lipopolysaccharide (10 μg/mL, 24 h) to mimic pro−inflammatory conditions. Bars represent the mean ± standard error of the mean (SEM) of at least n = 6 replicates, with individual data points indicated by circles (−LPS) and squares (+LPS). Statistical analysis was assessed using Kruskal–Wallis non−parametric analysis of variance followed by Dunn’s post hoc test. Comparisons were made versus the Fsk−treated, LPS−unstimulated group, whose mean value is represented by the black dashed line. Asterisks (*) indicate statistically significant differences (* *p* < 0.05). (**B**–**H**) Representative plots showing the mean square displacement (msd) versus time interval (tau) for fluorescent beads embedded in ASF samples under each experimental condition: 0.1% DMSO (**B**), 20 μM Fsk (**C**), 1 μM VX770 (**D**), 5 μM VX661 (**E**), 5 μM VX445 (**F**), 5 μM VX661 + 5 μM VX445 (**G**), and 30 μM PPQ102 (**H**). Black lines represent the average msd traces. For each treatment, the plots on the left illustrate experiments performed without LPS, and the plots on the right represent conditions with LPS stimulation (+LPS). The insets show the trajectory of a bead acquired during the MPT analysis.

The rheological properties of the ASF secreted by CFPAC-1 epithelial layers were evaluated using the multiple particle tracking (MPT) technique [37,38,39,40,41,42]. In samples treated with DMSO or Fsk, the recorded microviscosity values were markedly elevated (2.75 ± 0.03, n = 4 and 2.62 ± 0.06, n = 5, respectively), which is indicative of a dense and poorly hydrated fluid layer covering the apical surface. Figure 5 and Table S6 report the quantitative data of ASF microviscosity under the different treatment conditions, with Table S6 also presenting the statistical comparisons versus the DMSO-treated, LPS-unstimulated control group, together with the corresponding *p*-values. Treatment with the CFTR potentiator VX770 alone did not significantly alter microviscosity (2.61 ± 0.01, n = 5), suggesting that enhancement of channel gating alone is insufficient to restore fluid homeostasis in the absence of prior correction of CFTR trafficking to the apical membrane (see Figure 5 and Table S6). In contrast, treatment with CFTR correctors VX661 and VX445, administered either individually or in combination, led to a significant reduction in ASF microviscosity (Figure 5 and Table S6). This effect reflects improved hydration of the epithelial surface, likely due to enhanced CFTR rescue and function. This facilitates more effective apical surface fluid clearance. Among the treatments tested, the VX661 + VX445 combination elicited the most significant decrease in microviscosity, indicating a synergistic corrective effect (1.83 ± 0.02, n = 5, 1.69 ± 0.02, n = 4 and 1.45 ± 0.03, n = 4, for VX661, VX445 and VX661 + VX445, respectively). Notably, the beneficial effects of CFTR correctors on ASF microviscosity were preserved under inflammatory conditions induced by LPS preconditioning. This suggests that the efficacy of CFTR modulators is not substantially impaired by the pro-inflammatory environment (see Figure 5 and Table S5). In contrast, treatment with the selective CFTR inhibitor PPQ102 significantly increased ASF microviscosity under both basal and LPS-stimulated conditions (2.89 ± 0.11, n = 5 and 2.73 ± 0.14, n = 5, respectively), restoring values to levels comparable to or even higher than those observed in DMSO controls (see Figure 5 and Table S6). These findings confirm that the rheological improvements of ASF observed following modulator treatment are directly attributable to restored CFTR activity.

## 4. Discussion

Cystic fibrosis (CF) is a multisystemic disorder caused by mutations in the CFTR gene, leading to defective chloride and bicarbonate transport across epithelial surfaces. CFTR defects result in dehydrated, viscous secretions and progressive injury in different organs [1,2,3,4,5]. While lung disease is the leading cause of morbidity and mortality, the pancreas is the second most severely affected organ in CF. Ductal obstruction, fibrosis, and eventual fibrotic degeneration frequently culminate in exocrine pancreatic insufficiency, affecting the majority of individuals with CF [13,14]. Despite its clinical relevance, pancreatic involvement in CF has received comparatively less attention in in vitro studies than airway pathology.

The advent of CFTR modulators, small molecules that enhance CFTR protein folding, trafficking, and function, has revolutionized the management of CF [67]. In particular, the triple combination therapy comprising the potentiator ivacaftor (VX770) and the correctors tezacaftor (VX661) and elexacaftor (VX445), commercially known as Trikafta in the US and Kaftrio in Europe, has led to substantial clinical benefits. These include marked improvements in lung function, nutritional status, and quality of life, especially in individuals carrying at least one copy of the F508del mutation [10,68,69]. Despite these advances, the majority of preclinical research on these transformative therapies has concentrated on airway-derived epithelial models, leaving their effects on extra-pulmonary tissues, such as the pancreas, less thoroughly explored.

To address this knowledge gap, we used a multidisciplinary approach to investigate the effects of CFTR modulators VX770, VX661, and VX445 in an in vitro model of the pancreatic duct epithelium. We utilized CFPAC-1 cells, a human pancreatic ductal cell line derived from a CF patient that endogenously expresses the F508del CFTR mutation [33]. These cells retain several key characteristics of pancreatic ductal epithelium, including polarization [34,35], active ion and fluid transport [34,35,36,37], and mucin secretion [52]. Our primary aim was to determine whether the treatment with the CFTR modulators could restore critical physiological parameters in this CF pancreatic epithelial model, including transepithelial conductance (ΔTEC), fluid absorption (Jw), apical surface fluid pH, protein concentration, and microviscosity. Additionally, we aimed to evaluate whether a pro-inflammatory micro-environment could influence the efficacy of these modulators. In line with their distinct mechanisms of action, Fsk and VX770 were applied acutely, 10 min before measurements, to both the apical and basolateral compartments, whereas the correctors VX661 and VX445 (alone or in combination) were administered for 24 h prior to functional assays. This protocol reflects an established in vitro experimental approach whereby correctors, which act on CFTR folding and trafficking, require prolonged exposure, whereas the potentiator VX770, which exerts its effect on the gating properties of already membrane-localized CFTR, is effective following short-term application. Although this regimen does not directly mirror the chronic systemic administration of the triple therapy in patients, it is widely adopted in in vitro models to dissect individual drug effects on CFTR function [70,71].

Consistent with its role as a potentiator, VX770 alone produced minimal functional effects in the absence of prior CFTR correction. In contrast, the correctors VX661 and VX445 significantly improved CFTR-dependent ion transport, fluid reabsorption, apical surface fluid pH and microviscosity (see Figure 1, Figure 2, Figure 3, Figure 4 and Figure 5). Notably, the combination of VX661, VX445, and the acutely applied VX770 in the presence of Fsk yielded the most pronounced functional rescue across all measured parameters. However, it is important to clarify that these maximal effects likely reflect the full potentiating capacity of CFTR under the specific in vitro conditions employed, wherein both VX770 and Fsk were applied acutely. As such, this experimental design does not allow for a precise dissection of the individual contributions of Trikafta components and forskolin to the observed improvements. Further studies will be required to address this limitation. Nevertheless, the present results demonstrate that mutant CFTR expressed in pancreatic epithelial layers is pharmacologically responsive to modulators, similarly to what has been reported in airway models [47,72,73,74,75].

To simulate the chronic inflammatory environment typically found in the lungs of CF patients [76,77,78], human bronchial epithelial (HBE) in vitro models have been exposed to different stimuli, including lipopolysaccharide (LPS) [79], IFN-γ [80], TNF-α [81], interleukin-17 (IL-17) [82,83] bronchoalveolar lavage fluid (BALF) from pediatric CF patients, and supernatant from mucopurulent material (SMM) collected from explanted CF lungs [84,85]. Under these conditions, epithelial integrity and transepithelial electrical resistance are maintained, thus validating the models’ suitability for pharmacological testing. Notably, inflammatory conditioning has been shown to consistently enhance the efficacy of CFTR modulator combinations in restoring F508del-CFTR function [68,84,85,86].

Although the pancreas is traditionally considered a sterile organ, there is increasing evidence that CF-related ductal obstruction and epithelial injury can trigger localized inflammation, even in the absence of infection [87,88]. In pancreatic-sufficient (PS) patients, who account for approximately 10–15% of the CF population, episodes of acute or recurrent pancreatitis have been reported. By contrast, patients with pancreatic insufficiency (PI) tend to develop early and irreversible ductal fibrosis, which limits overt inflammatory manifestations [13,14,17,89]. Nevertheless, innate immune signaling likely contributes to disease progression in both subtypes [17,24,25,26,89]. To model the inflammatory aspect of CF pancreatic pathology in vitro, we exposed CFPAC-1 pancreatic ductal epithelial cell layers to 10 µg/mL LPS [30,31,32,90,91,92]. This concentration was selected based on preliminary experiments and on previous studies in epithelial and other mammalian cell models, demonstrating that 10 µg/mL LPS effectively induces a measurable inflammatory response, as evidenced by increased cytokine expression, without compromising cell viability [39,40,41,42,43,44].

Although LPS stimulation did not replicate infection per se, it served as a valuable strategy for simulating the pro-inflammatory microenvironment that may emerge in the CF pancreas in vitro.

Preconditioning with LPS did not reduce the therapeutic responses to VX770, VX661, or VX445. Conversely, we observed a trend towards improved outcomes, including increased ion transport, enhanced fluid absorption, a greater apical surface fluid pH alkalinization, and reduced ASF microviscosity (see Figure 1, Figure 2, Figure 3, Figure 4 and Figure 5). These results mirror those observed in HBE models, in which inflammation enhances the functional rescue of F508del-CFTR, suggesting that pro-inflammatory stress may improve modulator efficacy in the pancreatic context [68,84,85,93,94,95]. Importantly, the observed enhancement in modulator efficacy under pro-inflammatory conditions may be mechanistically linked to changes in the cellular proteostasis network induced by LPS exposure. Indeed, LPS preconditioning in CFPAC-1 cells could facilitate CFTR folding and trafficking via inflammation-induced modulation of the ER environment, which would consequently improve the response to correctors’ therapy.

Western blot analysis revealed that treating CFPAC-1 epithelial layers with CFTR correctors significantly increased the total expression and maturation rate of F508del CFTR (see Figure 3). These findings, particularly evident with the VX661 + VX445 combination, are consistent with those reported in HBE cells bearing the F508del mutation, in which corrector therapy leads to robust CFTR rescue and functional chloride channel activity at the apical membrane [10,73,96,97,98]. Interestingly, treatment with VX661 and VX445, especially in combination, resulted in an increased intensity of the immature band B, which contributes to the enhancement in total CFTR protein level. This may indicate a modulatory effect of correctors not only on CFTR processing and trafficking but also on its synthesis and/or post-translational stability. While this phenomenon has been rarely reported in airway epithelial models [71,73], where the primary action of correctors is generally limited to trafficking enhancement, our findings raise the possibility of a distinct modulatory response in pancreatic ductal cells. This implies that CFTR modulators may exert cell-type-specific effects in their mechanism of action. Further mechanistic studies will be required to clarify whether the observed increase in immature CFTR reflects enhanced translation, reduced proteasomal degradation, or other stabilizing mechanisms.

Importantly, pro-inflammatory preconditioning with LPS did not reduce the efficacy of the modulator treatment in rescuing F508del CFTR expression. On the contrary, we observed a slight yet consistent increase in total protein and maturation rate of mutant CFTR protein with VX661 and VX445, and an even greater enhancement with their combination. These results are consistent with earlier studies in F508del HBE epithelia, where exposure to inflammatory stimuli, such as supernatant from mucopurulent material (SMM), was shown to increase the endoplasmic reticulum (ER) protein folding capacity and promote CFTR processing [99,100]. It has been proposed that inflammation-associated ER expansion and up-regulation of molecular chaperones in airway epithelial cells may create a more favorable environment for the folding of conformationally unstable proteins such as F508del-CFTR. Furthermore, exposure to a pro-inflammatory stimulus such as LPS has been shown to enhance the activity of the ER quality control system, contributing to an accelerated degradation and recycling of mutant CFTR in epithelial cells. By extending these observations to a pancreatic epithelial model, our results suggest that similar mechanisms may operate beyond the airways. LPS preconditioning in CFPAC-1 cells could facilitate CFTR folding and trafficking via inflammation-induced modulation of the ER environment, which would consequently improve the response to correctors’ therapy.

TLR4, the canonical receptor for LPS, is expressed in the pancreatic ductal epithelium and activation of this pathway can trigger the secretion of cytokines [51,52,90,91,92]. In our study, LPS treatment resulted in a moderate, yet consistent, increase in the expression levels of pro-inflammatory cytokines IL-6, IL-8, and IL-1β (~1.5-fold, see Figure 4A–F). Although the responses observed in our model are not as robust as those seen in co-culture or airway-derived systems, the increase in IL-6, IL-8, and IL-1β expression confirms the activation of the pro-inflammatory pathways and validates the use of LPS as a stimulus for studying inflammation-modulated epithelial physiology in the pancreatic context [29,30,31].

In our experimental conditions, involving pre-exposure to 10 μg/mL LPS for 24 h, treatment with CFTR modulators, whether as single agents or in combination, did not reduce cytokine expression, suggesting that these drugs lack direct anti-inflammatory activity (see Figure 4A–F). These findings are consistent with those of previous studies that used primary human bronchial epithelial (HBE) cells expressing the F508del-CFTR, in which correctors and potentiators, including VX770/VX809 and VX770/VX661/VX445, failed to decrease cytokine levels following LPS or other inflammatory stimuli [101,102]. A few studies have reported partial reductions in the expression of pro-inflammatory cytokines, such as IL-8 or TNF-α, following CFTR modulator treatment [103]. However, such effects were often modest, and their reproducibility was limited, likely reflecting the influence of several experimental variables, including the timing and nature of the inflammatory challenge, the presence or absence of immune cells, and the cellular origin of the epithelial model employed. We acknowledge that our results should be interpreted in the context of a pre-established pro-inflammatory stimulation. Further studies exploring co-treatment protocols, different stages of inflammatory activation, LPS concentrations, and exposure durations will be instrumental in more precisely evaluating the immunomodulatory potential of CFTR modulators in pancreatic epithelial cells. Moreover, the absence of a measurable anti-inflammatory response in our study underscores the complexity of CF-related inflammation and supports the notion that restoring CFTR function alone may be insufficient to suppress epithelial cytokine production in the absence of immune-epithelial interactions. With all due limitations, our findings may have potential clinical relevance, as they suggest that adjunctive anti-inflammatory therapies could still be required to fully address the inflammatory burden in extra-pulmonary tissues such as the pancreas.

In summary, our findings reinforce the notion that CFTR modulators primarily act by rescuing defective F508del CFTR protein functional expression and restoring CFTR-mediated ion transport. Under the current in vitro conditions, which lack immune or stromal cell components, no measurable intrinsic anti-inflammatory activity was detected. Further studies are warranted to determine whether CFTR correction may indirectly influence inflammatory responses through crosstalk with immune cells in more complex co-culture systems or in vivo models.

Dot blot analysis was used to explore the mucin expression profile of CFPAC-1 epithelial preparations cultured in ALI under basal conditions (i.e., in the absence of pharmacological CFTR treatment or pro-inflammatory LPS stimulation). Although this approach was inherently qualitative and did not allow for rigorous quantification, it served to phenotypically characterize the secretory output of the epithelium. Among the mucins tested, MUC1, but not MUC5B, was detected in ASF samples (see Figure S2). This result is consistent with the established expression patterns of these macromolecules: MUC1 is a transmembrane mucin highly expressed in pancreatic ductal epithelium, whereas MUC5B is predominantly secreted by goblet cells in the respiratory and salivary glands [104]. This finding supports the physiological relevance of the CFPAC-1 system beyond CFTR expression alone and provides the basis for investigating mucus-related aspects of CF pathophysiology in the pancreatic context. Moreover, future studies assessing whether MUC1 secretion is modulated by CFTR correction could further elucidate the interplay between CFTR function and the secretory behavior CFPAC-1 epithelial preparations. This would be particularly relevant in light of our MPT data, which demonstrated that treatment with CFTR modulators reduced ASF microviscosity, potentially reflecting improved mucin unfolding and surface hydration. Such mechanisms are consistent with the known role of CFTR-mediated chloride and bicarbonate secretion in regulating mucus rheology and could help explain how CFTR correction contributes to restoring the biophysical properties of epithelial secretions.

As mentioned above, we employed multiple particle tracking (MPT) to assess how the pharmacological modulation of CFTR affects the rheological behavior of the ASF [45,46,47,49,50]. Our results demonstrated that treatment with CFTR correctors reduced ASF microviscosity, indicating improved fluid hydration and decreased viscoelastic resistance. Among the tested conditions, the combination of VX661 and VX445 yielded the most pronounced improvement, suggesting a synergistic effect in restoring surface fluid properties. Notably, these rheological improvements were sustained or slightly enhanced under inflammatory conditions.

These findings underscore the capacity of CFTR modulators to not only restore ion transport but also improve the physical characteristics of epithelial secretions. This dual action is particularly relevant for preventing mucus stasis and ductal obstruction in the pancreatic context of CF. Although the present study did not quantify mucin secretion or hydration status directly, the observed changes in ASF viscosity likely reflect enhanced CFTR-mediated water and bicarbonate transport. Future comparative analyses with other pancreatic epithelial models and complementary assessments of mucin abundance and fluid content would undoubtedly strengthen the mechanistic understanding of this phenomenon. 

## 5. Conclusions

This study demonstrates the effectiveness of VX661 and VX445, both individually and in combination, in addressing multiple aspects of F508del CFTR dysfunction in CFPAC-1 pancreatic epithelial cells. Inflammatory preconditioning with LPS does not reduce the efficacy of these CFTR modulators and may even amplify their functional impact. The results obtained support the use of Trikafta/Kaftrio also in the treatment of pancreatic symptoms of CF and confirm that ALI/LLI-cultured CFPAC-1 cells are a reliable and flexible in vitro model for the study of CFTR rescue and epithelial physiology in the context of the pancreas. Importantly, further validation in primary pancreatic tissues and in vivo systems will be necessary to confirm the physiological relevance and translational applicability of the obtained results and to better define the strengths and limitations of CFPAC-1 cells as a preclinical model for CF-related pancreatic disease.

## Figures and Tables

**Table 1:** Total protein concentration in the ASF of CFPAC-1 epithelial layers following treatment with CFTR modulators, in the absence or presence of LPS. Total protein concentration in the ASF was measured using the Bradford assay and expressed in μg/μL. The CFPAC-1 layers were treated for 24 h with the following: 0.1% DMSO (vehicle control), 20 μM Fsk, 1 μM VX770, 5 μM VX661, 5 μM VX445, a combination of 5 μM VX661 + 5 μM VX445, or the CFTR inhibitor PPQ102 (30 μM). This was done in the absence (−LPS) or presence (+LPS) of lipopolysaccharide (LPS, 10 μg/mL, for 24 h). Data are reported as mean ± SEM, with the number of independent replicates indicated in parentheses. Statistical comparisons were performed using the Kruskal–Wallis ANOVA followed by Dunn’s post hoc test. Comparisons were made versus the Fsk-treated, LPS-unstimulated group. Asterisks (*) denote statistically significant differences (*p* < 0.05).

	DMSO	Fsk	VX770	VX661	VX445	VX661 + VX445	PPQ102
−LPS	1.29	1.36	1.40	1.28	1.28	1.44	1.31
	±	±	±	±	±	±	±
	29.79	20.21	42.26	37.38	39.63	29.50	39.65
	(5)	(5)	(5)	(5)	(4)	(5)	(5)
+LPS	1.78	1.68	1.83	1.78	1.97	1.87	1.72
	±	±	±	±	±	±	±
	59.96	22.53	44.23	31.22	40.29	19.60	27.83
	(4)	(6)	(4)	(4)	(4)	(5)	(4)
	*	*	*	*	*	*	*

## Data Availability

The data presented in this study are available on request from the corresponding author.

## Supplementary Materials

The following supporting information can be downloaded at https://www.mdpi.com/article/10.3390/life15081315/s1, Table S1. ΔTEC (ΔG, μS/cm^2^) measurements under basal and pro-inflammatory conditions in CFPAC-1 epithelial layers. Table S2. Fluid secretion rate (J, μL·h-1·cm^−2^) in CFPAC-1 epithelial layers treated with CFTR modulators under basal and pro-inflammatory conditions. Table S3. pH of the airway surface fluid (ASF) in CFPAC-1 epithelial preparations treated with CFTR modulators under basal and pro-inflammatory conditions. Table S4. Evaluation of endogenous F508del CFTR protein expression in whole-cell lysates from CFPAC-1 epithelial layers. Table S5. Evaluation of IL-6, and IL-8 and IL-1β expression in the basolateral medium of CFPAC-1 epithelial layers. Table S6. Evaluation of the microviscosity of the apical surface fluid in CFPAC-1 epithelial layers under CFTR modulator treatment and pro-inflammatory conditions. Figure S1. Representative Coomassie blue-stained SDS-PAGE gels from concentrated basolateral medium of ALI-cultured CFPAC 1 epithelial layers. Figure S2. Representative dot blot assays showing detection of MUC1 (upper panel) and MUC5B (lower panel) in apical fluid collected from ALI-cultured CFPAC-1 epithelial layers (spots on the left), ALI-cultured Calu-3 epithelia (middle spots), and culture medium from HEK-293 cells (spots on the right).
