# Supplementary material for: CFTR Modulators Counteract F508del CFTR Functional Defects in a Pancreatic Epithelial Model of Cystic Fibrosis
Source: Life (Basel). 2025 Aug 19;15(8):1315. doi: 10.3390/life15081315 (PMC12387387; doi:10.3390/life15081315)
Supplement: Supplementary file 1 [file life-15-01315-s001.zip › life-3728813-supplementary.pdf]

## Supplementary data

**Table S1**. *ΔTEC (ΔG, µS/cm²) measurements under basal and pro-inflammatory conditions in CFPAC-1 epithelial layers.* Cells were treated for 24 hours with tezacaftor (VX661, 5 µM), elexacaftor (VX445, 5 µM), or their combination (VX661, 5 µM + VX445, 5 µM), applied basolaterally. Ten minutes before measurements, epithelial preparations (except those treated with DMSO) were acutely stimulated with forskolin (Fsk, 20 µM) and ivacaftor (VX770, 1 µM). Pro-inflammatory conditions were induced by basolateral exposure to lipopolysaccharide (LPS, 10 µg/mL) for 24 hours, with or without CFTR modulators. In CFTR inhibition assays, the inhibitor PPQ102 (30 µM) was applied 10 minutes after forskolin + VX770 stimulation. Data are expressed as mean ± standard error of the mean (SEM); n indicates the number of independent biological replicates. Statistical significance was evaluated using Kruskal–Wallis non-parametric ANOVA followed by Dunn's post hoc test ($p < 0.05$ considered significant). The last column reports *p* values for each condition compared to the forskolin-treated, non-inflamed control (Fsk), used as the reference condition.

| | | | **ΔTEC ( ΔG, µS/cm²)** | | |
|---|---|---|---|---|---|
| | **LPS** | **MEAN** | **SEM** | **n** | **p** |
| **DMSO** | - | 30.68 | 13.45 | 12 | 0.161 |
| | + | 56.74 | 18.33 | 12 | 0.753 |
| **Fsk** | - | 78.19 | 27.25 | 8 | - |
| | + | 98.23 | 25.16 | 12 | 0.293 |
| **VX770** | - | 199.42 | 20.4 | 12 | 0.308 |
| | + | 188.41 | 34.65 | 12 | 0.293 |
| **VX661** | - | 498.64 | 40.97 | 11 | 0.002 |
| | + | 635.21 | 26.2 | 12 | 0.001 |
| **VX445** | - | 544.36 | 28.26 | 10 | 0.002 |
| | + | 673.59 | 27.68 | 12 | 0.003 |
| **VX445 + VX661** | - | 844.42 | 39.4 | 12 | 0.004 |
| | + | 877.87 | 30.43 | 12 | 0.009 |
| **PPQ102** | - | -298.05 | 42.12 | 8 | 0.075 |
| | + | -383.24 | 33.37 | 12 | 0.085 |

**Table S2.** *Fluid secretion rate (J, μL·h$^{-1}$·cm$^{-2}$) in CFPAC-1 epithelial layers treated with CFTR modulators under basal and pro-inflammatory conditions.* CFPAC-1 epithelial layers were treated for 24 hours with tezacaftor (VX661, 5 μM), elexacaftor (VX445, 5 μM), or their combination (VX661, 5 μM + VX445, 5 μM), applied to the basolateral compartment. Ten minutes prior to measurement, epithelial preparations (except those treated with DMSO) were acutely stimulated with forskolin (20 μM) and ivacaftor (VX770, 1 μM). LPS (10 μg/mL) was added to the basolateral medium to induce a pro-inflammatory response. In CFTR inhibition assays, PPQ102 (30 μM) was applied following forskolin + VX770 stimulation to inhibit CFTR function. Data are presented as mean ± standard error of the mean (SEM). *n* represents the number of biological replicates. Statistical comparisons were performed using Kruskal–Wallis ANOVA followed by Dunn's post hoc test. A p-value < 0.05 was considered statistically significant. The last column reports *p* values for each condition compared to the Fsk-treated, LPS-unstimulated group, used as the reference condition.

| | | | **J (μL·h$^{-1}$·cm$^{-2}$)** | | |
|---|---|---|---|---|---|
| | **LPS** | **MEAN** | **SEM** | **n** | **p** |
| **DMSO** | - | 1.10 | 0.10 | 9 | 0,689 |
| | + | 1.18 | 0.07 | 10 | 0,758 |
| **Fsk** | - | 1.04 | 0.12 | 8 | - |
| | + | 1.22 | 0.06 | 10 | 0,216 |
| **VX770** | - | 1.10 | 0.09 | 10 | 0,747 |
| | + | 1.20 | 0.07 | 10 | 0,279 |
| **VX661** | - | 0.65 | 0.05 | 8 | 0,039 |
| | + | 0.60 | 0.05 | 12 | 0,006 |
| **VX445** | - | 0.62 | 0.06 | 10 | 0,008 |
| | + | 0.57 | 0.08 | 12 | 0,003 |
| **VX445 + VX661** | - | 0.58 | 0.09 | 12 | 0,001 |
| | + | 0.52 | 0.06 | 12 | 0,004 |
| **PPQ102** | - | 1.12 | 0.09 | 6 | 0,606 |
| | + | 1.22 | 0.06 | 9 | 0,271 |

**Table S3**. *pH of the airway surface fluid (ASF) in CFPAC-1 epithelial preparations treated with CFTR modulators under basal and pro-inflammatory conditions.*

The cells were treated with VX661 (5 µM), VX445 (5 µM), or their combination (VX661, 5 µM + VX445, 5 µM) for 24 hours. Ten minutes prior to sample collection, forskolin (Fsk, 20 µM) and VX770 (1 µM) were applied to both compartments. Pro-inflammatory conditions were induced by a 24-hour basolateral exposure to LPS (10 µg/mL). In CFTR inhibition assays, PPQ102 (30 µM) was added after forskolin/VX770 stimulation. Data are presented as mean ± standard error of the mean (SEM). *n* represents the number of biological replicates. Statistical comparisons were performed using Kruskal–Wallis ANOVA followed by Dunn's post hoc test. A p-value < 0.05 was considered statistically significant. The last column reports *p* values for each condition compared to the Fsk-treated, LPS-unstimulated group, used as the reference condition.

| | | pH | | | |
|---|---|---|---|---|---|
| | **LPS** | **MEAN** | **SEM** | **n** | **p** |
| **DMSO** | - | 7.16 | 0.01 | 8 | 0,116 |
| | + | 7.21 | 0.03 | 8 | 0,199 |
| **Fsk** | - | 7.14 | 0.04 | 8 | - |
| | + | 7.23 | 0.04 | 12 | 0,209 |
| **VX770** | - | 7.14 | 0.08 | 12 | 0,694 |
| | + | 7.22 | 0.04 | 11 | 0,378 |
| **VX661** | - | 7.4 | 0.03 | 8 | 0,016 |
| | + | 7.4 | 0.05 | 8 | 0,017 |
| **VX445** | - | 7.45 | 0.04 | 12 | 0,007 |
| | + | 7.41 | 0.05 | 12 | 0,006 |
| **VX445 + VX661** | - | 7.44 | 0.03 | 11 | 0,010 |
| | + | 7.44 | 0.03 | 12 | 0,006 |
| **PPQ102** | - | 7.15 | 0.03 | 7 | 0,326 |
| | + | 7.23 | 0.04 | 6 | 0,170 |

**Table S4**. *Evaluation of endogenous F508del CFTR protein expression in whole-cell lysates from CFPAC-1 epithelial layers.* The epithelial preparations were treated for 24 hours with CFTR correctors tezacaftor (VX661, 5 µM), elexacaftor (VX445, 5 µM), or their combination (VX661, 5 µM + VX445, 5 µM), applied to the basolateral compartment. Forskolin (Fsk, 20 µM) and ivacaftor (VX770, 1 µM) were applied to both the apical and basolateral sides. In selected groups, CFTR inhibition was achieved using PPQ102 (30 µM), and pro-inflammatory conditions were induced by a 24-hour exposure to lipopolysaccharide (LPS, 10 µg/mL). Western blot bands corresponding to total CFTR protein (sum of bands B + C) and maturation rate (ratio of the mature C-band to the total CFTR (C/(C+B))) were quantified by densitometric analysis using ImageJ software. Band intensities were normalized to the housekeeping protein actin and expressed relative to Fsk-treated samples without LPS (set as 1). Data represent the mean ± SEM (standard error of the mean) of at least four replicates. Statistical significance was determined using the Kruskal–Wallis ANOVA followed by Dunn's post hoc test. Comparisons were made versus the Fsk-treated, LPS-unstimulated group. The exact p-values are shown in the last column, with values of *p < 0.05 being considered statistically significant.

| | | F508del CFTR Total protein (C + B bands) | | | |
|---|---|---|---|---|---|
| | **LPS** | **MEAN** | **SEM** | **n** | **p** |
| **DMSO** | - | 1.01 | 0.07 | 4 | 0.167 |
| | + | 1.02 | 0.06 | 4 | 0.086 |
| **Fsk** | - | 1.00 | 0.06 | 5 | - |
| | + | 1.02 | 0.07 | 5 | 1.000 |
| **VX770** | - | 0.94 | 0.07 | 5 | 0.112 |
| | + | 1.09 | 0.07 | 5 | 0.106 |
| **VX661** | - | 1.86 | 0.09 | 4 | 0.012 |
| | + | 2.29 | 0.09 | 4 | 0.006 |
| **VX445** | - | 2.08 | 0.14 | 4 | 0.012 |
| | + | 2.53 | 0.14 | 4 | 0.002 |
| **VX445 + VX661** | - | 2.76 | 0.15 | 5 | 0.012 |
| | + | 3.06 | 0.15 | 5 | 0.006 |
| **PPQ102** | - | 0.97 | 0.08 | 4 | 0.086 |
| | + | 1.04 | 0.10 | 4 | 0.072 |

| | | F508del CFTR maturation rate (C/(C + B) bands) | | | |
|---|---|---|---|---|---|
| | **LPS** | **MEAN** | **SEM** | **n** | **p** |
| **DMSO** | - | 1.03 | 0.05 | 4 | 0.116 |
| | + | 1.01 | 0.08 | 4 | 0.198 |
| **Fsk** | - | 1.01 | 0.14 | 5 | - |
| | + | 1 | 0.12 | 5 | 0.223 |
| **VX770** | - | 0.99 | 0.15 | 5 | 0.205 |
| | + | 0.99 | 0.17 | 5 | 0.472 |
| **VX661** | - | 2.33 | 0.08 | 4 | 0.006 |
| | + | 2.54 | 0.1 | 4 | 0.002 |
| **VX445** | - | 2.28 | 0.13 | 4 | 0.012 |
| | + | 2.53 | 0.12 | 4 | 0.003 |
| **VX445 + VX661** | - | 2.46 | 0.13 | 5 | 0.012 |
| | + | 2.66 | 0.13 | 5 | 0.006 |
| **PPQ102** | - | 1.04 | 0.07 | 4 | 0.198 |
| | + | 1.04 | 0.08 | 4 | 0.512 |

**Table S5.** *Evaluation of IL-6, and IL-8 and IL-1β expression in the basolateral medium of CFPAC-1 epithelial layers.* Epithelial preparations were treated for 24 hours with CFTR correctors tezacaftor (VX661, 5 μM), elexacaftor (VX445, 5 μM), or their combination (VX661, 5 μM + VX445, 5 μM), applied to the basolateral compartment. Ten minutes prior to sample collection, forskolin (Fsk, 20 μM) and ivacaftor (VX770, 1 μM) were applied to both the apical and basolateral sides. In selected groups, CFTR inhibition was achieved using PPQ102 (30 μM), and pro-inflammatory conditions were induced by a 24-hour exposure to lipopolysaccharide (LPS, 10 μg/mL). Cytokine levels in the basolateral medium were assessed by Western blot analysis. To ensure comparability across lanes, total protein loading was verified by Coomassie blue staining of the PVDF membranes used for IL-6 and IL-8 detection. Bands' intensities were quantified by densitometric analysis using ImageJ software. Band intensities were quantified by densitometric analysis using ImageJ software and normalized to total protein levels. Values are expressed relative to Fsk-treated samples without LPS (set as 1). Data represent the mean ± SEM (standard error of the mean) of at least four replicates. Statistical significance was determined using Kruskal–Wallis ANOVA followed by Dunn's post hoc test. Comparisons were made versus the Fsk-treated, LPS-unstimulated group. The exact p-values are indicated in the last column, with values of *p < 0.05 being considered statistically significant.

| | | IL-6 | | IL-8 | | IL-1β | |
|---|---|---|---|---|---|---|---|
| LPS | | MEAN ± SEM (n) | p | MEAN ± SEM (n) | p | MEAN ± SEM (n) | p |
| DMSO | - | 1.02 ± 0.03 (4) | 0.086 | 0.95 ± 0.04 (4) | 0.072 | 0.95 ± 0.09 (4) | 0.197 |
| | + | 1.46 ± 0.04 (4) | 0.012 | 1.50 ± 0.04 (4) | 0.012 | 1.49 ± 0.07 (4) | 0.003 |
| Fsk | - | 1.00 ± 0.03 (5) | - | 1.00 ± 0.04 (5) | - | 1.00 ± 0.11 (5) | - |
| | + | 1.45 ± 0.06 (5) | 0.012 | 1.56 ± 0.05 (5) | 0.031 | 1.49 ± 0.08 (5) | 0.02 |
| VX770 | - | 1.09 ± 0.04 (4) | 0.166 | 0.95 ± 0.04 (4) | 0.065 | 1.06 ± 0.09 (4) | 0.223 |
| | + | 1.37 ± 0.06 (4) | 0.005 | 1.47 ± 0.08 (4) | 0.022 | 1.37 ± 0.07 (4) | 0.005 |
| VX661 | - | 1.00 ± 0.04 (4) | 0.072 | 0.95 ± 0.03 (5) | 0.087 | 0.96 ± 0.09 (5) | 0.174 |
| | + | 1.47 ± 0.05 (4) | 0.005 | 1.51 ± 0.06 (4) | 0.012 | 1.39 ± 0.07 (4) | 0.004 |
| VX445 | - | 1.09 ± 0.03 (4) | 0.193 | 0.93 ± 0.07 (4) | 0.086 | 0.96 ± 0.07 (4) | 0.275 |
| | + | 1.48 ± 0.05 (4) | 0.004 | 1.53 ± 0.09 (4) | 0.001 | 1.34 ± 0.06 (4) | 0.009 |
| VX445 + VX661 | - | 1.04 ± 0.02 (4) | 0.123 | 0.94 ± 0.06 (5) | 0.084 | 0.97 ± 0.08 (5) | 0.378 |
| | + | 1.44 ± 0.05 (4) | 0.012 | 1.56 ± 0.09 (5) | 0.003 | 1.31 ± 0.08 (5) | 0.002 |
| PPQ102 | - | 1.10 ± 0.04 (4) | 1.000 | 1.05 ± 0.04 (5) | 0.259 | 0.94 ± 0.08 (5) | 0.535 |
| | + | 1.42 ± 0.04 (4) | 0.007 | 1.41 ± 0.08 (5) | 0.002 | 1.32 ± 0.08 (5) | 0.008 |

**Table S6.** *Evaluation of the microviscosity of the apical surface fluid in CFPAC-1 epithelial layers under CFTR modulator treatment and pro-inflammatory conditions.* Cells were treated for 24 hours with CFTR correctors tezacaftor (VX661, 5 µM), elexacaftor (VX445, 5 µM), or their combination (VX661, 5 µM + VX445, 5 µM), applied to the basolateral compartment. Ten minutes prior to ASF collection, forskolin (Fsk, 20 µM) and ivacaftor (VX770, 1 µM) were applied to both the apical and basolateral sides. In selected groups, CFTR inhibition was achieved using PPQ102 (30 µM), and pro-inflammatory conditions were induced by a 24-hour exposure to lipopolysaccharide (LPS, 10 µg/mL). Microviscosity was analyzed using the multitracker plugin of the ImageJ software, followed by analysis with IgorPro 9 software. Data are expressed as mean ± standard error of the mean (SEM), and *n* indicates the number of independent biological replicates per condition. Statistical comparisons were performed using Kruskal–Wallis ANOVA followed by Dunn's post hoc test. Comparisons were made versus the Fsk-treated, LPS-unstimulated group. The exact p-values are indicated in the last column, with values of *p < 0.05 being considered statistically significant.

| | | Microviscosity (cPoise) | | | |
|---|---|---|---|---|---|
| | **LPS** | **MEAN** | **SEM** | **n** | **p** |
| **DMSO** | - | 2.75 | 0.03 | 4 | 0,402 |
| | + | 2.71 | 0.05 | 5 | 1,000 |
| **Fsk** | - | 2.62 | 0.06 | 5 | - |
| | + | 2.56 | 0.20 | 5 | 0,091 |
| **VX770** | - | 2.61 | 0.01 | 5 | 0,187 |
| | + | 2.47 | 0.06 | 5 | 0,084 |
| **VX661** | - | 1.83 | 0.02 | 5 | 0,005 |
| | + | 1.75 | 0.03 | 5 | 0,005 |
| **VX445** | - | 1.69 | 0.02 | 4 | 0,014 |
| | + | 1.56 | 0.04 | 5 | 0,011 |
| **VX445 + VX661** | - | 1.45 | 0.03 | 4 | 0,002 |
| | + | 1.34 | 0.05 | 5 | 0,030 |
| **PPQ102** | - | 2.89 | 0.11 | 5 | 1,000 |
| | + | 2.73 | 0.14 | 5 | 0,187 |

**Supplementary Figures**

**Figure S1**

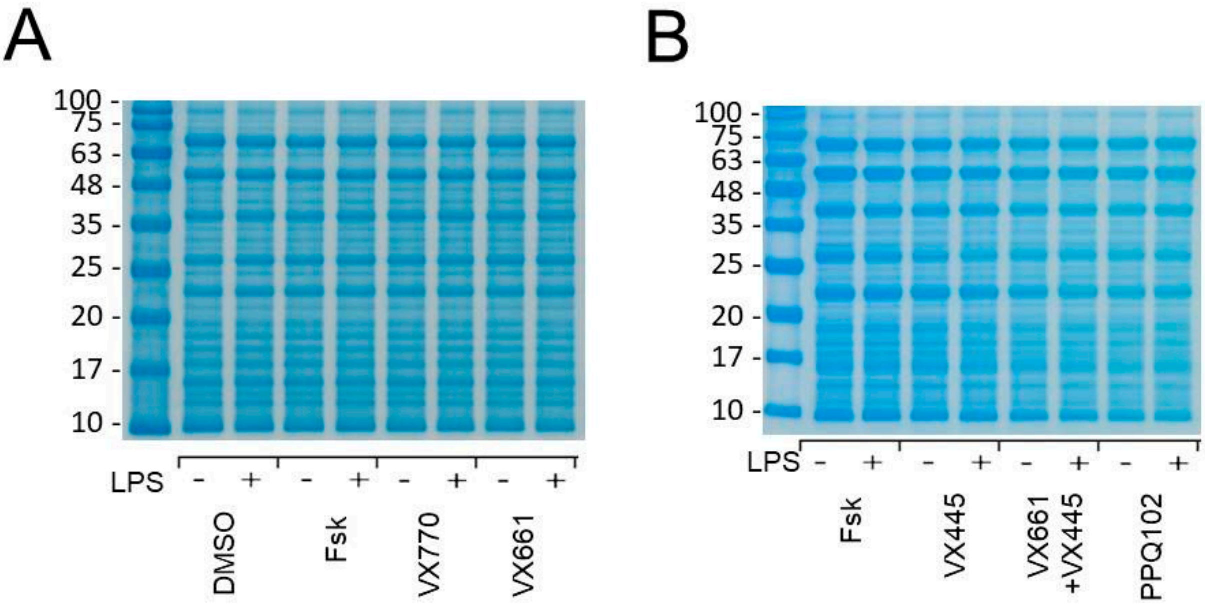


**Figure S1.** Representative Coomassie blue-stained SDS-PAGE gels from concentrated basolateral medium of ALI-cultured CFPAC-1 epithelial layers. (**A**) From left: vehicle (0.1% DMSO), forskolin (Fsk, 20 µM), VX770 (1 µM), and VX661 (5 µM). (**B**) From left: forskolin (Fsk, 20 µM), VX445 (5 µM), VX661 (5 µM) + VX445 (5 µM) combination, and PPQ102 (30 µM), in the absence or presence of LPS preconditioning. Coomassie staining was performed to visualise total protein patterns and was used as a loading control to verify uniform protein loading across lanes. The densitometric signal from each membrane was used for normalisation of cytokine band intensities in the corresponding Western blot analyses, enabling semi-quantitative comparison of expression levels between treatment groups. Molecular weight markers (kDa) are indicated to the left of each membrane image**.**

**Figure S2**

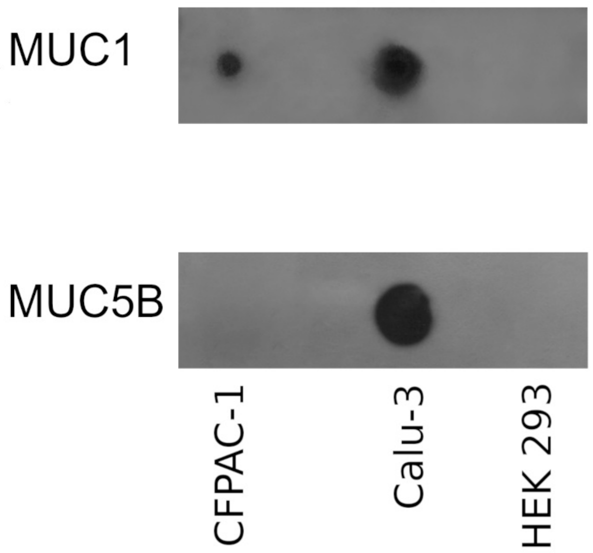


**Figure S2.** Representative dot blot assays showing detection of MUC1 (upper panel) and MUC5B (lower panel) in apical fluid collected from ALI-cultured CFPAC-1 epithelial layers (spots on the left), ALI-cultured Calu-3 epithelia (middle spots), and culture medium from HEK-293 cells (spots on the right). For each sample, 10 µL (approximately 2 µg/µL) of the sample was applied to nitrocellulose membranes and probed with the indicated antibodies. Dot blots were repeated on at least three independent preparations per cell type.
